# Host-microbiota interactions shaping T-cell response and tolerance in type 1 diabetes

**DOI:** 10.3389/fimmu.2022.974178

**Published:** 2022-08-18

**Authors:** Shubhabrata Majumdar, Yong Lin, Matthew L. Bettini

**Affiliations:** ^1^ Immunology Graduate Program, Baylor College of Medicine, Houston, TX, United States; ^2^ Department of Pathology, University of Utah, Salt Lake City, UT, United States

**Keywords:** mimicry, T1D (type 1 diabetes), microbiota, T-cell, Th-17, Treg cells, tolerance

## Abstract

Type-1 Diabetes (T1D) is a complex polygenic autoimmune disorder involving T-cell driven beta-cell destruction leading to hyperglycemia. There is no cure for T1D and patients rely on exogenous insulin administration for disease management. T1D is associated with specific disease susceptible alleles. However, the predisposition to disease development is not solely predicted by them. This is best exemplified by the observation that a monozygotic twin has just a 35% chance of developing T1D after their twin’s diagnosis. This makes a strong case for environmental triggers playing an important role in T1D incidence. Multiple studies indicate that commensal gut microbiota and environmental factors that alter their composition might exacerbate or protect against T1D onset. In this review, we discuss recent literature highlighting microbial species associated with T1D. We explore mechanistic studies which propose how some of these microbial species can modulate adaptive immune responses in T1D, with an emphasis on T-cell responses. We cover topics ranging from gut-thymus and gut-pancreas communication, microbial regulation of peripheral tolerance, to molecular mimicry of islet antigens by microbial peptides. In light of the accumulating evidence on commensal influences in neonatal thymocyte development, we also speculate on the link between molecular mimicry and thymic selection in the context of T1D pathogenesis. Finally, we explore how these observations could inform future therapeutic approaches in this disease.

## Introduction

Type 1 Diabetes (T1D) is a chronic autoimmune disorder resulting from T cell-mediated destruction of the insulin-producing β cells found in the pancreatic islets (1). Like many other autoimmune disorders, T1D is caused by the combined influence of genetic predisposition and environmental factors. More than 50 genetic loci have been linked to an increased risk of developing T1D (2, 3). Not every at-risk individual will become diabetic however, and environmental factors appear to play a significant role in T1D development (4). This can be best demonstrated in monozygotic twins where a twin has approximately 35% chance of developing T1D if the other sibling is affected (5–7). Similarly, in the genetically identical non-obese diabetic (NOD) mouse strain, not all mice develop autoimmune diabetes (8). Furthermore, the incidence of T1D has increased dramatically over the past decades, strongly indicating that environmental factors are accelerating the development of T1D (9).

Similar increase in other autoimmune and inflammatory disorders is also observed (10). The general improvement of living standards over the past century is believed to have contributed to the increased incidence of these disorders. Termed the “hygiene hypothesis”, it is believed that “clean” environments lead to improper training of the developing immune system in early life, resulting in aberrant immune responses to foreign- and self-components (11). Hence, much research has gone into how various environmental and lifestyle factors, such as birthing method, diet, infection, and antibiotic use contribute to the risk for developing T1D. The results of these efforts have pointed to the alterations in the gut microbiota as a critical variable in whether a genetically susceptible individual will go on to develop T1D (4).

In this review, we will discuss the current understanding of how alterations in gut microbiota result in diabetogenic T cell activation and T1D. We first discuss factors in healthy gut development, followed by a summary of findings from human studies that identified common features of gut alteration associated with increased or decreased risk of developing T1D. We then move onto findings in animal studies, where precise manipulations in the gut microbiota and the immune system provided mechanistic insights into how gut microbial alterations result in diabetogenic T cell activation and autoimmune diabetes. As microbial antigens can share similar epitopes with diabetogenic autoantigens, we also discuss the potential role of microbial antigens in shaping diabetogenic T cell development in light of the reported link between gut microbiota and thymic selection. Furthermore, we discuss potential therapeutic interventions based on these findings and what work still needs to be done to fill in the gap in knowledge. Finally, we propose distinguishing genetic and environmental factors within the emerging concept of T1D endotypes, where there can be multiple different sets of etiological pathways resulting in T1D (12).

## Association of early life microbial gut colonization with T1D

The first three years of life are crucial for the establishment of a healthy gut microbiome. This early life period undergoes waves of microbial colonization which gradually diversify and stabilize into a mature adult microbiome. After analyzing stool sample 16s rRNA sequences collected from European and US children as part of The Environmental Determinants of Diabetes in the Young (TEDDY) study, *Stewart et al.* have identified three distinct phases of microbiome progression: a developmental phase (3-4 mo. age), a transitional phase (15-30 mo. age) and a stable phase (≥31 mo. age) (13). Immediately after birth, the intestinal environment favors the initial expansion of facultative anaerobes like *Staphylococcus*, *Streptococcus*, *Escherichia coli*, and *Enterobacteria* that are fast colonizers and good survivors in an oxygen rich environment (14, 15). In the developmental phase, with the introduction of maternal milk, the infant gut is dominated by members of the Actinobacteria and Firmicutes phyla as they are better able to utilize and survive on milk oligosaccharides. *Bifidobacterium* spp. were the dominant taxa at the genus level during this time. At the species level, *B. bifidum, B. breve, B. dentium, Lactobacillus rhamnosus and Staphylococcus epidermidis* were observed at higher levels in breast-fed individuals (13). When compared across different geographical regions, the microbial profiles are very similar during this early period with breast milk being the major diet source (16). With the introduction of solid food after around 4-6 months age and the subsequent reduction in breast-feeding, there is a gradual increase in microbial diversity and richness. Microbial taxa feeding on milk oligosaccharides become less dominant and allow for the diversification of the microbiome with different taxa that are able to utilize nutrients supplied by the local diet (13, 16, 17). Functional metagenomic studies show a decline in enzymes like L-lactate dehydrogenase involved in milk fermentation and a corresponding increase in fiber metabolism genes like transketolase (17). By the third year, the microbiome matures and reaches a more stable state with a high alpha diversity and dominance by members of the Firmicutes phylum (13).

Several studies in humans and rodent T1D models have suggested that the microbiota is altered in T1D patients in comparison to healthy individuals. T1D patients seem to have a lower Firmicutes/Bacteroidetes ratio and reduced microbial diversity (18–20). Higher levels of *Bacteroides* species have been reported in T1D cases across different studies on Finnish, Spanish and US populations (18, 19, 21). Pre-diabetic children seropositive for autoimmune antibodies also had higher levels of *Bacteroides* (22). Concomitantly, there is an observed reduction in the abundance of short-chain fatty acid (SCFA) producing bacteria in T1D patients. Lower levels of Firmicutes and Actinobacteria members have been reported in T1D cases including, *Lactobacillus* and *Bifidobacterium* species which are dominant in breast-fed children (19, 21, 23). In accordance with this observation, some studies have suggested that infants that have not been breast-fed are at a higher risk of developing T1D than breast-fed infants, although there is no strong association with duration of breastfeeding (24–26). T1D patients also have lower levels of *Clostridium* cluster IV and XIVa members that are known to produce SCFAs (18). Lower levels of *Prevotella*, which are good SCFA producers and found abundantly in populations on a plant-based diet, have also been reported in T1D cases (19, 21). Although these observations describe altered microbiota in T1D patients, they may not be indicative of causation and might be a consequence of the altered immune system under autoimmune conditions. To address the issue of causation, longitudinal studies were required to look at the microbiota of healthy T1D genetically susceptible and pre-diabetic individuals from early time points. One such study analyzed stool samples from a cohort of 33 Finnish and Estonian infants genetically pre-disposed to T1D recruited as part of the Pathogenesis of Type 1 Diabetes - Testing the Hygiene Hypothesis (DIABIMMUNE) study. They observed a significant reduction in alpha-diversity of microbial species in infants who developed T1D with a trending overabundance of pathobiont containing genera like *Blautia, Ruminococcus and Streptococcus* (20). In the TEDDY study, healthy subjects had more *Lactobacillus rhamnosus*, *Bifidobacterium dentium, Streptococcus thermophilus and Lactococcus lactis* whereas sero-converters and T1D patients had higher *Streptococcus* spp.*, Bifidobacterium pseudocatenulatum, Roseburia hominis, Alistipes shahii* and *Bacteroides vulgatus* (17). Higher levels of *Parabacteroides* spp. levels and lower *Akkermansia* spp. levels were also reported in T1D patients recruited in the TEDDY study (13). **Table 1** provides a summary of important commensal bacteria that are associated with T1D incidence along with their proposed mechanisms.

## Maintenance of gut-barrier integrity and its role in T1D

One of the major functions of the host immune-microbial cross-talk is maintenance of the gut barrier integrity. Loss of gut barrier integrity can lead to bacterial translocation and leakage of gut luminal products into sterile immune compartments causing inflammation and non-homeostatic activation of the host immune system. In order to prevent this, the host immune system has coevolved with beneficial gut microbiota to maintain homeostatic conditions where the luminal side of the barrier can harbor commensal microbiota while maintaining controlled exchange of luminal products across the epithelium. The barrier site is stratified into different levels of protection and can be divided into an outer luminal mucus layer and an inner intestinal epithelial barrier (IEB) (46). The mucus layer acts as a buffer zone for harboring microbial communities while preventing their direct contact with the epithelial layer (47). This layer is primarily composed of mucins that are special glycoproteins with O-linked glycans secreted by the intestinal goblet cells (48). The mucus layer also contains a milieu of antimicrobial peptides (AMPs) like defensins, cathelicidins and RegIIIγ that directly keep microbial communities in check and prevent their contact with the epithelium (46). These peptides are produced by the intestinal epithelial cells (IECs) (49). The immune cells are located along the epithelium in the lamina propria and in specialized lymphoid structures called Peyer’s patches. Intestinal dendritic cells (DCs) can randomly sample bacterial components near the epithelium and interact with B and T-cells in the Peyer’s patches to induce IgA production (46, 50). IgA are transcytosed across the epithelium and bind to specific bacteria to prevent their translocation across the barrier (50). The composition of the mucus layer acts as a source of nutrition for the microbiota, and along with the AMPs shape the microbial communities. The microbiota, through their cell wall components and metabolites, provide feedback to the host, predominantly in a MyD88 and inflammasome dependent manner (51, 52). This influences the production of tight junction proteins, mucins and AMPs by the IECs.

Early loss of gut barrier integrity with low-grade intestinal inflammation has been observed in T1D diagnosed cases (53). It has been suggested to be a contributing factor to the initiation of autoimmune pathogenesis as studies have shown that loss of gut barrier integrity precedes T1D onset. In T1D susceptible patients with islet autoimmunity, increased intestinal permeability was observed before clinical onset of diabetes (54). Loss of gut barrier integrity has been shown to occur prior to insulitis in the spontaneous autoimmune diabetes model in BioBreeding (BB) rats (55, 56). The increased permeability is attributed to disassembly of tight junctions through upregulation of zonulin (57, 58). Loss of barrier integrity is also associated with altered architecture of the mucus layer as seen in the NOD mice. It involved decreased expression of immune-regulatory mucins Muc1 and Muc3 and increased inflammatory Muc4 (59). This can be attributed to a reduction of SCFA producing intestinal bacteria in T1D patients as SCFAs like butyrate have been shown to enhance tight junction formation and mucin production in the epithelial cells (17, 18, 22, 60–62). Butyrate production as result of metabolic cooperation between *Bacteroides thetaiotaomicron* and *Faecalibacterium prausnitzii* (*Clostridium* cluster IV) also impacted goblet cell maturation and mucin glycosylation in gnotobiotic rodents (33). Acetate produced by *Bifidobacterium longum infantis* was involved in protecting mice from *E. coli* O157:H7 enteropathogenesis by preventing the translocation of Shiga toxin across the IEB (39). Similarly, *Bifidobacterium dentium* was also shown to modify goblet cells and increase mucin synthesis through acetate production (41).

Production of AMPs like cathelicidins is altered in NOD mice and T1D patients (63, 64). Cathelicidins are constitutively produced by intestinal epithelial cells and their expression is restricted to the pre-weaning period in mice (65). Interestingly, pancreatic endocrine cells also express Cathelicidin-related antimicrobial peptide (CRAMP) under the influence of gut microbiota (64). Defects in CRAMP production were observed both in the pancreas and colon of NOD mice (63, 64). Defective CRAMP production was shown to be associated with altered intestinal microbiota, enhanced Type I interferon expression and pathological imprinting of the immune system. Early life CRAMP treatment increased beneficial bacteria in the gut, reduced Type I interferon levels and reduced diabetes incidence (63). Similarly, the RegIIIγ has also been implicated to have a protective role in T1D (66). RegIIIγ is produced by intestinal Paneth cells under the influence of Toll-like receptor (TLR) signaling. *Akkermansia muciniphila* mediated upregulation of RegIIIγ and mucin production has been suggested as a possible mechanism for T1D protection in NOD mice (42, 43). *Bifidobacterium breve*, which has a negative correlation with T1D incidence, was also shown to enhance RegIIIγ expression in a TLR dependent manner (13, 17, 40).

Microbiota can strengthen gut barrier function through cross-talk with the innate immune system. This has been elucidated in mice colonized with segmented filamented bacteria (SFB). SFB induces IL-23 expression in CX3CR1+ myeloid cells leading to IL-22 secretion by ILC3s in the lamina propria (30). IL-22 is known for its role in promoting epithelial cell growth, AMP and mucin production (67). This was one of the proposed explanations for a study where SFB colonization in NOD mice showed protection from diabetes development (31). Along the same lines, a recent study reported low levels of IL-23 and IL-22 production as a result of intestinal inflammation in T1D mouse models. In parallel, there was an observed reduction in SFB abundance and loss of gut integrity with decreased expression of tight junction proteins and Muc2 (32).

The studies highlighted in this section provide an understanding of the importance of gut-barrier integrity maintenance in T1D. Gut commensals that are negatively associated with T1D incidences, play an important role in the maintenance of gut barrier integrity through regulation of tight junction proteins, mucin formation, AMP production and modulation of the host mucosal immune cells.

## Loss of gut-barrier integrity and the establishment of an inflammatory gut environment

As mentioned earlier, T1D is a polygenic autoimmune disorder. Among the genetic loci conferring the highest risk are those localized in the HLA genes (68). While various components of the immune system are known to play a role in this disease, T1D is considered a T-cell-mediated disorder where activated diabetogenic T cells directly result in the destruction of islet β cells. The association between gut dysbiosis and T1D is well established, but how the commensal changes influence the immune environment within the gut that eventually result in the activation of the diabetogenic T cell population has only recently been investigated. Much insight has been gained with various T1D animal models such as the spontaneously diabetic NOD mouse model, streptozotocin (STZ)-induced diabetic mouse model, and the BB rat. In this section, we will discuss the current understanding of how dysbiosis and the compromised epithelial barrier establishes an inflammatory gut environment.

As the activation of T cells depends on the presentation of the cognate antigens by professional antigen-presenting cells in an inflammatory context, recent work has highlighted the role of the gut innate immune system in breaking T cell tolerance against islet beta cells during gut dysbiosis. Using various knockout animal models, it has been shown that innate signaling proteins such as MyD88 and TRIF, which control microbe-sensing through pattern-recognition receptors, can greatly impact diabetes incidence (69, 70). MyD88 deficiency protected the NOD mouse almost completely from diabetes by 30 weeks of age (69). Interestingly, when MyD88 deficiency was generated in germ-free mouse, the diabetes rate was similar to wild-type NOD mouse. These observations pointed to two important insights: 1. autoimmune diabetes is genetically determined in the NOD mouse, and 2. microbiota-immune interactions determine the rate at which diabetes occurs. The latter forms the basis of the balanced signal hypothesis, which suggests that immune-microbiota interaction induces both pro-inflammatory and anti-inflammatory signals and that the relative strength of each signal affects the disease risk. Indeed, TLR’s influence on diabetes incidence is receptor specific. TLR2 knockout protected the NOD mice from diabetes, but the protection was reversed in the germ-free setting, indicating that TLR2 interaction with the microbiota promotes diabetes (71). In contrast, targeting signaling through TLR4 appeared to provide protection from diabetes in this model (71). To complicate matters further, subtypes of the TLR4 ligand LPS (E. coli-derived vs Bacteroides-derived) possess either stimulatory or inhibitory activity when bound to TLR4 and may have opposing effects on T1D risk (72). Thus, interactions between gut microbiota and the innate immune system, mediated by various pattern recognition receptors, influences the immune context in which autoreactive T cells see its cognate antigen and may accelerate or delay disease. These findings suggest that in dysbiosis, the changing microbial diversity in the gut may shift the balance of signals toward a pro-inflammatory state, leading to compromised gut integrity. Infiltration of gut microbes through the leaky gut then activates the innate immune cells residing below the epithelium, thereby establishes an inflammatory environment in which diabetogenic T cells are likely to be activated. Interestingly, a study looking at a large cohort of Korean patients suggested that individuals with Crohn’s disease, characterized by gut dysbiosis and inflammation, are at a higher risk of developing T1D (73). 

Neutrophils are a subset of innate granulocytes critically important in early immune responses to inflammation and infection. It stands to reason that neutrophils would be involved in the early immune response to invading gut commensal in the leaky gut. Current data point to the involvement of neutrophils in early stages of islet autoimmunity. Recently diagnosed T1D patients exhibited a reduction of neutrophils in circulation (74–76). This observation was associated with the accumulation of neutrophils in the pancreas, suggesting a role for neutrophils in the pathogenesis in T1D (74). In agreement, a separate study detected elevated levels of the neutrophil chemoattractant IL-8 in the pancreas of T1D patients (77). Additionally, elevated levels of circulating neutrophil granule enzymes (neutrophil elastase and proteinase 3) were detected in recently diagnosed T1D patients and in autoantibody-negative at-risk individuals (78). The same study also found that elevated circulating neutrophil granule enzymes were closely associated with increased neutrophil extracellular traps (NET) formation, suggesting the involvement of NETosis in T1D pathogenesis. Similar results were obtained in a large scale study in which NET-forming neutrophils were identified in the pancreas of T1D patients and pre-symptomatic at-risk individuals (79). Collectively, these data indicate neutrophils are involved in the early stages of human T1D. In contrast, another study did not find increases in serum levels of neutrophil elastase and proteinase 3 although the study subjects were diagnosed with T1D within three years, as opposed to within one year in the other aforementioned studies (80). This difference in criteria for patient inclusion may explain the discrepancy between the studies.

More recent work also identified an association between NET production and leaky gut in newly diagnosed T1D patients (81). Six of the eight markers of NET production examined in this study showed an increase in T1D patients, and protein arginine deiminase type 4 (PAD4) levels in particular appear to be the best predictor of gut leakage and T1D diagnosis. Furthermore, zonulin and LPS were also increased in T1D patients, indicating the association between leaky gut, infiltration of microbes and microbial products, and neutrophil activation. Neutrophil and NET formation have similarly been found in the NOD mouse model (82–85). Infiltration of neutrophils into the NOD pancreas can be detected as early as 2 weeks of age (82). In contrast, no neutrophil infiltration was detected in the B6 mouse strain which is not genetically susceptible for autoimmune diabetes. Importantly, a recent report described a direct link between gut leakage and NET formation where the induction of gut leakage by dextran sulfate sodium (DSS) treatment in the NOD mouse increased NET production and accelerated diabetes (83). However, in PAD4 knockout NOD mice, in which PAD4-mediated formation of NETs was blocked, diabetes incidence was dramatically reduced. Furthermore, T1D patient-derived NETs can induce the activation of dendritic cells (DCs), increasing its expression of HLA and co-stimulatory molecules as well as production of inflammatory cytokines (86). These changes would provide an ideal activating environment for autoreactive T cells in T1D.

Enteric infections are also associated with compromised gut barrier integrity and accelerated T1D (87, 88). *Citrobacter rodentium* (*C. rodentium*) infection is a colonic infection model that induces gut leakage. Infection with *C. rodentium* in the NOD and STZ-induced diabetes mouse model accelerated diabetes incidence (88). It was found that *C. rodentium* infection reduced antimicrobial peptide CRAMP production in the intestine, resulting in gut leakage and increased activation of macrophages and CD86+ cDCs in the mesenteric lymph node (mLN). In CRAMP knockout mice, *C. rodentium* infection further exacerbated the diabetes incidence.

**Figure 1:**
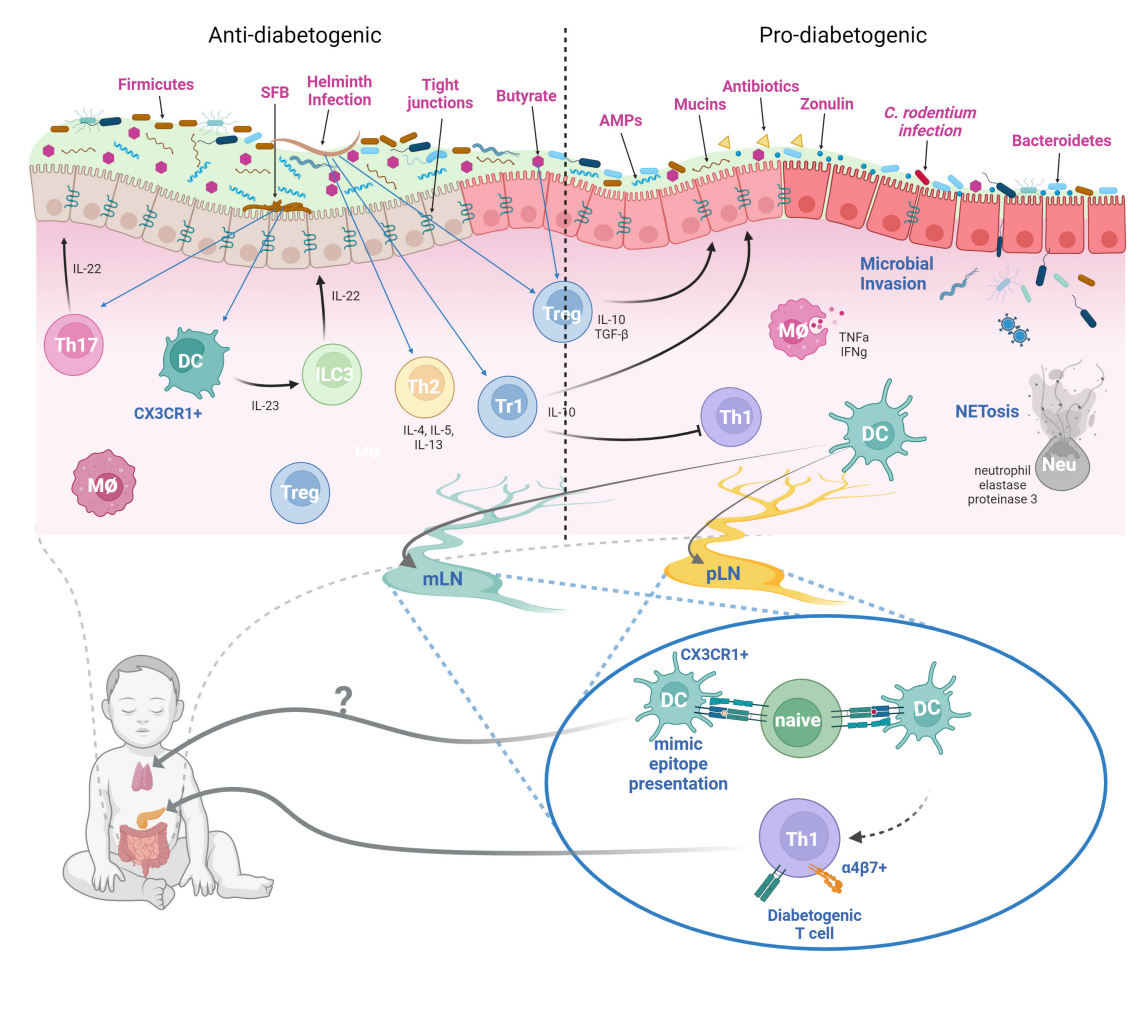
Dysbiosis, Compromised Gut Barrier, and T1D Risk. In a healthy gut microenvironment, most microbial species are not in direct contact with the epithelial barrier. The mucus layer serves as a barrier between the commensal microbes and the epithelium. Contained within the mucus layer are various innate immune components such as the antimicrobial peptides (AMPs) such as defensins, cathelicidins, and RegIIIγ. The adaptive immune system also supplies the mucus layer with IgA antibodies that bind to commensal microbes and prevent their adherence to the epithelium. However, certain commensals such as the SFB can directly adhere to the epithelium and stimulate Th17 response. SFB-induced immunity appears to protect NOD mice from autoimmune diabetes through enhancing intestinal barrier conferred by Type 3 immune cytokine IL-22. A high proportion of Firmicutes to Bacteroidetes ratio seems to protect against T1D presumably due to Firmicutes being a major butyrate producer. The SCFA butyrate can improve barrier integrity by enhancing barrier function of tight junctions, improve mucin production, and induction of intestinal Tregs. Helminth infections, which are more common prior to the improved hygienic practices in the past century, may also decrease T1D incidence through the induction of a type 2 response and the generation of regulatory Foxp3+ and Tr1 cells. In dysbiosis, altered Firmicute/Bacteroidetes ratio may diminish SCFA production, reducing intestinal barrier function, and promote leaky gut syndrome. Infections by certain microbes such as *C. rodentium* can also enhance gut permeability. Additionally, antibiotic usage can also reduce commensal diversity and lead to dysbiosis. The resulting depletion of the mucus layer and the compromised tight junction may allow infiltration of microbes through the epithelium and induce innate immune cell activation. Production of inflammatory cytokines by innate cells and NETs by neutrophils further exacerbates gut permeability. The inflammatory gut environment promotes the activation of diabetogenic T cells in the mLN and the pLN, which then traffic to the pancreas and cause islet damage. Furthermore, β-cell antigen mimics found in the gut microbes and enteric pathogens can also potentially activate diabetogenic T cells and contribute to disease. Whether these mimics can be trafficked to the thymus to mediate T cell central tolerance is unclear (figure created with BioRender.com).

Overall, these results suggest that homeostatic innate immune responses maintain a healthy gut environment and resists diabetes development, whereas innate immune response toward infiltrating microbiota due to compromised gut barrier promotes an inflammatory environment favorable to the activation of self-reactive lymphocytes (**Figure 1**).

## Gut-pancreas immune axis in T1D

While pancreatic lymph node (pLN) is critical for T1D development in the NOD mouse, it wasn’t clear whether other lymphoid tissues can also serve as activation site of diabetogenic T cells as well (89). Initial report suggesting mesenteric lymph node (mLN) as the initial site of diabetogenic T cell activation used adoptive transfer of lymphocytes from various lymphoid organs of NOD mice at different ages to determine which age and organ had the most diabetogenic potential (90). In the young donor mice (3-weeks old), it was found that lymphocytes with diabetogenic potential were found almost exclusively in the mLNs. In contrast, by 6-weeks old, most of the diabetogenic T cells are found in the pLNs, with mLN-derived lymphocytes exhibiting only intermediate diabetogenic potential. Additionally, the expression of the gut-homing integrin LPAM (α4β7 integrin) was found in islet-infiltrating lymphocytes, indicating gut mucosal origin (91). Furthermore, NOD mice treated at an early age with an antibody blocking the mucosal addressin cell adhesion molecule-1 (MAdCAM-1) were almost completely protected from diabetes at 32-weeks old (9% diabetic compared to 80% in the control group) (92). Thus, a link between mLN and the pancreas was established. Subsequent report further established that antigens derived from the GI tract are able to reach both the mLN and the pLN and activate their cognate T cells (93). Moreover, disruption of the gut barrier allowed for the translocation of bacteria to the pLN, which activated the innate receptor NOD2 in immune cells that contributed to diabetes in the STZ-induced diabetes model (94). Overall, these data indicate inflammatory gut environment can promote activation of diabetogenic T cells in the mLN and pLN, resulting in islet autoimmunity and diabetes (**Figure 1**).

## Regulation of T cell phenotype by the gut commensal microbiota

The interactions between the gut commensal microbiota, the epithelium, and the immune system promotes proper barrier function in steady state conditions. The commensals protect against colonization of pathogenic microbes through competition for space and nutrients but also strengthen the barrier integrity by influencing T cell differentiation (95). T cell response in the healthy gut is typically associated with the generation of homeostatic Th17 cells and Tregs, which can result in the production of various cytokines that enhance tissue barrier function. For example, the commensal SFB can adhere to the epithelium and trigger the secretion of serum amyloid A and result in IL-17 production by SFB-specific Th17 cells (30, 31, 96). IL-17 is known to act on the epithelium to enhance barrier integrity by controlling the localization of the tight junction protein occludin and enhancing production of AMPs (97–99). In contrast to homeostatic Th17 response, IL-23-driven Th17 response seems to promote autoinflammatory and autoimmune conditions such as seen in experimental autoimmune encephalomyelitis and inflammatory bowel disease. Data suggest the pathology is partly a result of IL-23 mediated conversion of Th17 to Th1 cells (100).

Gut commensals also drive Treg development. The intestinal Treg population is enriched for clones that recognize commensal microbiota (101, 102). In agreement, germ-free mice given commensal microbiota showed an expansion and activation of Tregs in the colonic lamina propria (103). In neonatal mice, the colonizing microbiota drives the formation of a population of Rorγt+ Tregs near weaning (104). The accumulation of these Tregs is associated with the dramatic shift in commensal population as a result of diet change from milk to solid food and the availability of dietary metabolites such as SCFAs and retinoic acid. These Tregs are critical to prevent the pathological imprinting of the immune system that increases susceptibility to colitis and allergic conditions (104). Microbiota can also regulate Treg development through their surface components and metabolites such as SCFAs. For example, capsular polysaccharide A (PSA) from the human commensal *Bacteroides fragilis* can stimulate dendritic cells to promote the differentiation of naïve T cells into IL-10-producing Tregs (105, 106). Feeding mice PSA enhanced generation of these Tregs and conferred protection against a mouse model of Multiple Sclerosis (107). Similarly, SCFAs generated from fermentation of dietary fibers has been shown to promote Treg generation in the gut (108–110). The SCFA butyrate appears to enhance Treg generation by enhancing histone H3 acetylation in the *Foxp3* promoter and the conserved non-coding regions (108, 110). Butyrate also suppresses inflammatory cytokine production in DCs, resulting in a more tolerogenic, Treg-promoting environment (111, 112). Other commensal derived SCFA propionate also promoted histone acetylation at the Foxp3 locus and result in Treg generation (108). Overall, these data indicate the significant role of commensal microbiota in influencing intestinal T cell phenotype. Given that β-cell specific T cells can be activated in gut-draining lymph nodes, it is very possible that these T cells are subjected to similar phenotypic regulation by the gut microbiota and the disruption of which may result in increased risk for T1D.

## Microbial regulation of thymocyte development

Central tolerance is established in the thymus during T-cell development. Developing CD4+ CD8+ double positive thymocytes are positively selected in the thymic cortex on the basis of their ability to recognizing non-agonist peptides in the context of host MHC presented by the cortical thymic epithelial cells (cTECs). Positively selected thymocytes mature into CD4+ and CD8+ single positive thymocytes and migrate into the thymic medulla where they undergo further selection mechanisms (113–115). In the medulla, thymocytes are exposed to a wide repertoire of self-antigens presented by medullary resident and migratory antigen-presenting cells (APCs). The medullary thymic epithelial cells (mTECs) can present tissue-restricted antigens in an autoimmune regulator (AIRE) and Fezf2 dependent manner (116, 117). Thymic resident CD8+ DCs can acquire antigens from mTECs and cross-present to developing CD8+ thymocytes (118). Migratory APCs including Sirpα+ CD11b+ cDC2s, CD11c+ B220+ CCR9+ pDCs and B-cells, can transport peripheral self-antigens to the thymus through blood circulation under homeostatic conditions (119–121). High affinity agonistic interactions of developing thymocytes with self-antigens in the thymus leads to deletion as a mechanism to weed out potentially auto-reactive T-cells (114, 122–124). In addition, as an extra layer of protection, developing CD4+ thymocytes with an intermediate affinity for self-antigens can be converted into FoxP3+ Treg cells that can suppress auto-reactivity in the periphery (124).

Central tolerance plays a major role in preventing autoimmunity as mutations in AIRE can lead to multi-organ autoimmunity termed as autoimmune polyendocrinopathy candidiasis ectodermal dystrophy (APECED). In T1D, there is a genetic pre-disposition to develop diabetes in individuals bearing specific high-risk HLA haplotypes and length of the variable number of tandem repeats (VNTRs) of the insulin promoter. HLA-DQ8 is one such MHC haplotype whose peptide binding groove is unfavorable for tight binding of insulin peptide B:9-23. On the other hand, individuals with shorter VNTRs might generate less insulin peptides for presentation to the developing thymocytes and lead to thymic escape of autoreactive T-cells (125–127). The pancreas also produces many post-translationally modified antigens like hybrid insulin peptides (HIPs) that might not be presented to the developing thymocytes. HIP activated T-cells have been identified in T1D patients and can initiate T1D pathogenesis as seen in NOD mice (128, 129). So, bolstering central tolerance by enhancing presentation of islet specific antigens in the thymus is one potential mechanism of tackling T1D.

Recent evidences suggest that intestinal microbiota can modulate T-cell selection in the thymus. TCR sequencing of intestinal and thymic Tregs in TCR^mini^ mice, revealed significant repertoire overlap and resulted in identification of microbiota specific Treg TCRs in the thymus and gut (102). Antibiotic treatment altered both the thymic and intestinal Treg repertoire, suggesting intestinal microbiota might influence thymic selection of microbiota specific Treg cells. In line with this observation, microbial colonization of neonatal mouse skin resulted in a wave of migration of microbial antigen specific Tregs into the skin that mediated immune tolerance in the skin (130). Transient FTY720 treatment in the neonatal time window prevented accumulation of the Tregs in the skin while there was an increased accumulation of these Tregs in the thymus. This indirectly suggested that microbial colonization of the skin might influence microbial antigen specific Treg development in the thymus. Interestingly, colonization of adult skin with microbiota or blocking Treg migration in the neonatal period, resulted in no immune tolerance to the microbial antigen. This suggested that there might be a specific neonatal time window when the commensal microbiota can influence thymic development of microbiota specific T-cells (130). Recently, we were able to detect microbial 16SrRNA signatures in the thymus and demonstrate migration of CX3CR1+ DCs from the gut to the thymus to induce expansion of SFB antigen specific CD4+ thymocytes in young SFB colonized mice but not in adult mice. The migration of CX3CR1+ DCs is dependent on the microbiota as antibiotic treatment showed a decrease in thymic DC subsets (131). In another study, colonic pDCs were shown to migrate into the neonatal thymus in a *Bacteroides fragilis* dependent manner and influence development of PLZF+ innate lymphocytes (132).

Thymic development of MAIT (mucosal associated invariant T) cells can also be controlled through microbial metabolites (133). MAIT cells are evolutionarily conserved T cells with an invariant TCRα (Vα7.2–Jα33 in humans, and Vα19–Jα33 in mice) chain that associates with a limited repertoire of β-chains. They recognize and respond to bacterial or fungal Vitamin B2 precursors presented on the MHC Class Ib molecule MR1 (134, 135). MAIT cells can be detected in blood, liver and various mucosal barrier tissues (134). MAIT cells respond and migrate into infected or inflamed tissues to control pathogen load through effector cytokines like IFNγ, TNFα and IL-17 and direct lysis of infected cells through granzyme B (134, 135). On the other hand, under homeostatic conditions, mucosal RORγt+ MAIT17 cells play an important role in maintenance of gut barrier integrity through the production of IL17 and IL22 (136). Germ-free mice bear a reduced number of RORγt+ MAIT17 cells with an overall skewing towards IFNγ production and reduced IL-17 production. Upon co-housing with SPF mice, MAIT17 cell numbers are rescued in a TCR-dependent manner. The effect was mediated through thymic capture and presentation of the bacterial vitamin B2 synthesis by-product 5-OP-RU [5-(2-oxopropylideneamino)-6-D-ribitylaminouracil (5-OP-RU)] when applied topically on mouse skin or when GF mice were colonized by a vitamin B2 proficient commensal strain *Enterococcus hirae* (133). This has potential implications for T1D, as RORγt+ MAIT17 cells have been shown to be important for maintenance of gut barrier integrity and play a protective role in T1D (136).

Overall, these observations provide support for a novel concept for the regulation of thymic T-cell development by commensal microflora. Although this concept has not been explored directly in the context of T1D yet, we speculate that the gut-thymus axis might play a role in T1D and other autoimmune disorders through the recruitment of microbiota specific thymic Tregs which are important for maintenance of tolerogenic conditions in the gut. The gut-thymus axis could also influence T1D pathogenesis by regulating the thymic development of islet specific cross-reactive T-cells through molecular mimicry.

## Molecular mimicry in T1D

Thymic selection is not foolproof and healthy individuals do have self-antigen specific T-cells in peripheral circulation and tissues (27, 137, 138). Under homeostasis, they do not initiate pathogenesis due to tolerogenic conditions maintained by Treg cells and a lack of pro-inflammatory cues (139). However, inflammatory conditions caused by infection or gut-barrier leakiness may cause a breakdown of tolerance, including enhanced antigen processing and presentation of APCs. In the previous sections, we have discussed how T1D patients are associated with microbial dysbiosis and a leaky gut that indirectly leads to activation of islet specific T-cells in the pLN (17, 54, 93, 94). Furthermore, gut microbial peptides may directly activate cross-reactive islet specific T-cells through structural and functional homology to islet specific antigens. This is termed as molecular mimicry. Molecular mimicry as a mechanism for T1D induction was proposed decades ago through the identification of sequence similarity of glutamate de-carboxylase (GAD), an enzyme produced by islet β-cells, and the P2C protein of the Coxsackie virus (140). It was suggested that T and B lymphocytes activated during the initial viral infection are able to cross-react with islet specific GAD and initiate β-cell destruction (141). Other viral molecular mimicry candidates as potential initiators for T1D had also been proposed including the human cytomegalovirus (hCMV), rotavirus, varicella and measles virus (142–144). Although these studies identify T-cell cross-reactivity to viral peptides, there is a no direct evidence showing the ability of viral molecular mimics to initiate T1D pathogenesis. Newer studies have also identified T1D specific molecular mimicry candidates among the gut commensal species. One particular study in NOD mice, identified an IGRP (islet-specific glucose-6-phosphatase catalytic subunit-related protein) peptide mimic in *Fusobacteria* expressing a magnesium transporter (Mgt). They were able to demonstrate activation of IGRP specific CD8 T-cells and initiation of diabetes *in vivo* upon transfer of T-cells activated by the mimic into NOD mice. Super-colonization by *Fusobacteria* resulted in accelerated diabetes in their NY8.3 TCR(tg) NOD mice (45). The cation efflux transporter ZnT8 was identified as an auto-antigen in T1D patients (145). Healthy individuals can have ZnT8 specific CD8+ T-cells in circulation and substantial fraction of them were shown to bear an antigen experienced phenotype that suggested cross-priming of these T-cells. A bacterial peptide mimic derived from *Bacteroides stercoris* was shown to be a mimic of the ZnT8_186-194_ peptide and was shown to cross-react with ZnT8_186-194_ specific T-cells. Interestingly, the mimic peptide had a stronger agonist potency than the ZnT8_186-194_ peptide (27). The gut commensal *Parabacteroides distasonis* has been positively correlated with T1D cases (13, 146). *P. distasonis* colonization in NOD mice has been demonstrated to accelerate diabetes. A molecular mimic of the insulin peptide B:9-23, derived from *P. distasonis*, previously identified in a DIABIMMUNE study, was shown to activate insulin specific T-cells *ex vivo* with similar potency (29, 147). An integrase derived peptide expressed by several *Bacteroides* species was found to encode a low avidity mimetope of IGRP_206-214_ that can recruit IGRP specific CD8+ T-cells to the GALTs and activate them. Surprisingly, in this study, these diabetogenic CD8+ T-cells recruited to the gut were able to suppress colitis through cytotoxic killing of gut DCs presenting the bacterial mimetope (28). Although not entirely focused on T1D, this study provides strong evidence for priming of cross-reactive diabetogenic T-cells in the GALTs. Overall, all these examples support the possibility of bacterial peptide mimics priming pathogenic autoreactive T-cells and accelerating T1D progression. However, more mechanistic evidence is required to support the role of molecular mimicry as an initiator of T1D pathogenesis.

## Environmental factors and T cell phenotype in T1D

### Chemical-induced gut leakage

As previously mentioned, DSS-driven gut leakage in the NOD mouse promotes the formation of NETs by neutrophils and accelerates diabetes (83). When co-cultured with autologous splenocytes, NETs drove the proliferation of memory Th1 and Th17 cells in the NOD mouse. Additionally, DSS-induced acceleration of T1D is associated with an increased the ratio of Th1-to-Th2 cells in the pLN. Th1 cytokines TNFα and IFNγ was also increased as a result of DSS-induced gut permeability. In contrast, the reduced gut permeability, NET formation, and diabetes incidence in PAD4 knockout mice was associated with reduced Th1-to-Th2 cell ratio. While β-cell specific T cell populations were not evaluated in this context, the general shift toward Th1 phenotype is consistent with the idea that autoimmune diabetes is a Th1-driven disease. In the same study, authors also found a dramatic increase in integrin α4β7+ T cells in the pLN, indicating a gut-primed origin. Importantly, dendritic cell activation by NETs derived from T1D patients also promoted the generation of IFNγ-producing Th1 cells (86).

### Infection

Infections have long been associated with T1D risk. Whether the infection promotes or inhibits disease depends on the type of infection. Enteric infection by *C. rodentium* in the NOD mouse resulted in enhanced gut permeability, inflammation, and accelerated diabetes (87, 88, 148). This change was associated with increased frequency of inflammatory M1 macrophages, activated cDCs, and IFNγ-producing CD4 Th1 and CD8 Tc1 cells (87, 88, 148). Consistent with previous finding, increase in α4β7+ CD4 and CD8 T cells were observed, indicating gut-associated T cell priming. Importantly, diabetogenic CD4 T cell clone BDC2.5 showed greater proliferation in the pLN when transferred into NOD mice pre-infected with *C. rodentium* (148). Similarly, *C. rodentium* infection in the NOD.8.3 transgenic mice that bears a diabetogenic CD8 T cell receptor specific for the islet antigen IGRP resulted in increased activation of the CD8 T cell population as indicated by increased CD44 expression (87, 148).

In contrast, various murine parasitic infections including *Helignosomoides polygyrus* (*H. polygyrus*)*, Strongyloides venezuelensis* (*S. venezuelensis*)*, Taenia crassiceps* (*T. crassiceps*), and *Schistosoma mansoni* (*S. mansoni.*) reduced the incidence of T1D in mice (149–152). As mentioned earlier, the ‘hygiene hypothesis’ posits that increased cleanliness and improved healthcare in developed countries is a major contributor to the increased incidence of various inflammatory and autoimmune conditions. One proposed mechanism of the ‘hygiene hypothesis’ is the reduced incidence of helminth infections (153). Helminth infection-induced type 2 immune responses are associated with the production of effector cytokines that enhance tissue barrier integrity. Additionally, helminth-induced peripheral Treg may have bystander effects that reduce risk for autoinflammatory and autoimmune diseases such as T1D (154–156). Infection with *S. mansoni* or treatment with *S. mansoni*-derived soluble extracts can provide protection from T1D in the NOD mouse (149, 157, 158). *S. mansoni*-derived ribonuclease ω-1 driven Th2 immune response and Treg generation appear to be at least in part responsible for the disease protection (159, 160). Additionally, *S. mansoni* proteins have been reported to reduce colonic inflammation caused by trinitrobenzene sulfonic acid, suggesting that *S. mansoni* may also modulate T1D through reducing intestinal inflammation and gut leakage (161). Likewise, infection of the parasite *S. venezuelensis* ameliorated insulitis and was associated with a Th2 polarized response as well as increased IL-5 and IL-10 expression in splenocytes (150). Infection by the parasite *H. polygyrus* similarly protected NOD mice from T1D through blocking a Th1 response and inducing a Th2 response (162). Surprisingly, in IL-4 knockout NOD mice, where Th2 response is blocked, *H. polygyrus* infection still conferred protection from diabetes (163). This was due to the production of IL-10 by β-cell-specific effector T cells in the infected mice. These data suggest *H. polygyrus* mediates protection of T1D through shifting from Th1 to Th2 response as well as induction of Tr1-like Tregs that are Foxp3-IL-10+. Overall, Helminth infections seem to confer protection from T1D through shifting the adaptive immune response and the production of immunosuppressive cytokines.

### Antibiotics

Accumulating evidence indicates alteration of gut microbiota through antibiotic usage affects T1D risk. In the NOD mouse, maternal antibiotic treatment had mixed results in the diabetes incidence of the offspring. In one study, treatment of the mother with a mixture of antibiotics (metronidazole, gentamicin, and polymyxin) accelerated diabetes incidence at 20 weeks (164). The treatment reduced gut microbiota diversity in the offspring and altered their T cell population. Specifically, frequency of CD8 T cells was increased in the mLN of the offspring from mother treated with antibiotics. CD4+CD62L- T cells also increased in the Peyer’s patch of the antibiotics group, suggesting enhanced T cell activation. Frequency of Foxp3+ T cells were unchanged. No further characterization of β-cell antigen-specific T cell responses were noted, therefore it is unknown whether the maternal antibiotics treatment affected the activation state of those cells. In contrast, another study found that treating NOD mothers with neomycin, polymyxin B, and streptomycin, conferred protection from diabetes in the offspring (165). The offspring’s gut microbiota showed decrease in *proteobacteria* and increase in G+ *Firmicutes* families *Lachnospiraceae* and *Coriobacteriacea*. In the immune compartment, the antibiotics group showed a higher frequency of tolerogenic APCs that resulted increased Foxp3+ Tregs and reduced T-bet+ and IFNγ+ CD4 and CD8 T cells. Importantly, co-culturing the BDC2.5 and NY8.3 diabetogenic T cell clones with the APCs from the antibiotics group reduced their activation and proliferation when compared to the controls. Similarly, co-transfer of NY8.3 T cells with the APCs into NOD.scid mouse demonstrated a delay in diabetes when the APCs were from the antibiotics-treated group. The differences in antibiotics used might explain the discrepancies seen in these two studies. Another possibility is that the baseline microbiota composition could be different between these two labs.

Studies on the effect of post-natal early life antibiotic treatment in the NOD mouse generally resulted in an acceleration of diabetes incidence (166–171). Comparison of unperturbed gut microbiota between the NOD mouse and the diabetes-resistant non-obese resistant (NOR) mouse showed that the NOD mouse naturally had less diabetes-protective bacteria (166). This difference was exacerbated with prolonged antibiotics treatment, which led to decreased in microbial diversity and protective species (eg. *Desulfovibrio* spp., *Prevotella* spp., *B. acidifaciens*). Furthermore, antibiotic treatment led to reduction in the metagenome of gene clusters involved in SCFA synthesis, which are associated with T1D protection (170). Immunologically, the gut alteration was associated with decreased IL-17+ CD4 T cells in the gut-lymphoid tissues. Interestingly, an increase in IFNγ+ CD4 T cells was seen only in Vancomycin-treated group and not in Neomycin-treated group, despite both groups exhibiting accelerated diabetes to similar degrees (166). Shorter-term therapeutic-dose antibiotic treatment likewise found accelerated diabetes in the antibiotics group (167, 169). Microbiota alterations resulted in reduced Bifidobacterium and the butyrate-producers *S24-7*, *Clostridiales*, *Lactobacillus*, *Oscillospira*, and *Ruminococcus* (167, 171, 172). In agreement, cecal butyrate levels were decreased with the short course of antibiotics (169). Dramatic downregulation of serum amyloid A, a known promoter of Th17 response, was noted in the ileal gene expression profile of the antibiotics group, consistent with the observed decreased of Th17 and Treg proportions in the lamina propria. Subsequent work supplementing Vancomycin antibiotics treatment with a butyrate-rich diet ameliorated the antibiotic-accelerated T1D and its associated induction of IFNγ+ Th1 and Tc1 cells (170). A different rescue approach *via* maternal cecal microbiota transfer to antibiotics-treated neonates also rescued the offspring from antibiotics-accelerated T1D and associated gut microbiota changes (171). Antibiotics treatment also decreased bacterial bile acid metabolites, which are important in the maintenance of RORγ+ Tregs in the gut (173).

### Diet

Various formulations have been studied in animal models to determine the properties of diabetogenic and anti-diabetogenic diets. In the diabetes-prone BB rat model, it was reported that rats fed a cereal diet exhibited a high rate of diabetes (174). In terms of T cell phenotype, cereal diet is associated with increased activation of CD3+ and CD8α+ lymphocytes in the gut and an increased ratio of *Ifng* to *Il4* mRNA. In contrast, rats fed a low-antigen hydrolyzed casein (HC) diet had approximately 50% reduction in diabetes incidence. Examination of the intestinal tissue identified an increase in CRAMP expression in the HC diet group when compared to the cereal diet group. CRAMP’s essential role in maintaining gut microbiota homeostasis may have contributed to the reduced diabetes incidence (175). Similar results were obtained in the diabetes-prone LEW.1AR1-iddm rat model when fed either the cereal or HC diet, with the HC diet group exhibiting significantly reduced diabetes and *Ifng* to *Il4* mRNA when compared to the cereal group (176).

## Therapeutic interventions

Based on the common features of gut commensal alterations seen in T1D, various approaches have been attempted to correct these deficits. General strategies include 1. replenishing or enhancing protective microbial species, 2. enhancing resistance to gut barrier disruption, 3. suppressing innate inflammatory response, and 4. deviating immune response toward Th-2/17 and Treg phenotypes. Modification of the gut microbiota by the probiotic VSL#3, which is enriched in the Lactobacillaceae family of bacteria, has shown benefits against T1D in the NOD mouse (177). The protective effect was mediated by an increase in indoleamine 2,3-dioxygenase (IDO) and IL-33 in the intestine. IDO is known for enhancing a tolerogenic phenotype in DCs which suppresses T cell activation while also promote Treg generation (178, 179). Intestinal IL-33 can drive production of TSLP and retinoic acid in intestinal epithelium, which are known to promote Treg generation (180–183). In addition, IL-33 can promote a Th2 immune response and may help shift the immune cells from the more diabetogenic Th1 response (184, 185). Supplementation with the probiotic IRT5 (*Lactobacillus acidophilus*, *Lactobacillus casei*, *Lactobacillus reuteri*, *Bifidobacterium bifidium*, and *Streptococcus thermophiles*) similarly saw a decrease in diabetes incidence (186). The effect was associated with reduced Th1 skewing and increased Treg frequency in the mLN and the lamina propria of the small intestine. Besides replenishing protective microbes through probiotic supplementation, the prebiotic approach aims at enhancing SCFA generation through increased consumption of dietary fiber such as inulin (187). Increased dietary fiber fermentation by intestinal microbes can then increase the SCFA output in the gut. In the NOD mouse and the STZ-induced diabetes model, inulin intake ameliorated diabetes (187). The inulin-treated group showed an overall decrease in CD4 T cell frequency in the mLN and a slight increase in the Treg frequency. Increased levels of AMPs α-defensin and RegIIIγ were also noted. As previously mentioned, AMPs are important in maintaining gut microbial stability and provide an additional barrier layer between the microbes and the gut epithelium, effectively enhancing barrier integrity (188). Supplementation with the yeast-derived prebiotic β-glucan also delayed diabetes and reduced insulitis (189). The shifting of the colonic immune system toward Th17 and Tregs appears to mediate the protection conferred by β-glucan. To suppress innate inflammatory response, one group tried to degrade T1D-promoting NETs in the intestine of NOD mice *via* oral administration of staphylococcal nuclease (SNase)-producing *Lactococcus lactis* (190). SNase treatment degraded NETs and reduced inflammation in the intestine and pancreas. IFNγ+ and Rorγt+ CD4 T cells were also reduced in the mLN and pLN. In addition, butyrate-producing bacteria *Oscillospira*, *Faecalibacterium* and *Ruminococcus* were increased. Finally, decrease in zonulin levels indicated improved gut barrier function with SNase treatment.

## Conclusion

The current data support the idea that gut commensal alteration is a central factor mediating environmental variables associated with developing T1D in at-risk individuals. Dysbiosis and enhanced gut permeability are associated with development of T1D in both humans and the NOD mouse model. Reduction of SCFA-producing bacteria species are generally associated with increased T1D risk. Indeed, particular diets and enteric pathogens known to decrease SCFA-producing species are associated higher risk of T1D development. Reduced availability of SCFAs such as butyrate, the main source of energy for colonocytes, likely impaired their ability to maintain barrier integrity as well as reduced generation of Tregs in the gut. Invasion of microbes into the tissue *via* compromised gut barrier results in immune cell activation in both the gut and the pancreas, leading to activation of the innate immune system and establishment of a pro-Th1 adaptive immune response in at-risk individuals. As summarized in this review, this plausible sequence of events leading to T1D is supported by the observation that enhancing gut barrier function through manipulation of the gut microbiota, suppression of neutrophil activation, or deviation of the adaptive immune response towards Th2/Th17/Treg all conferred protection against T1D. While this is a plausible sequence of events leading to T1D, lack in genetic diversity in animal models limits its translatability toward the human disease. Particularly, given the heterogeneity in human T1D, the relative importance of each step in the sequence with respect to the emerging concept of T1D endotypes is unclear (12).

It may be beneficial to distinguish genetics-driven endotypes and environment-driven endotypes. HLA variations confer the greatest genetic risk in T1D susceptibility. HLA-DR3 and HLA-DR4 are strongly associated with distinct islet autoantibodies, indicating potentially distinct genetics-driven endotypes(191, 192). While antibiotics can drive accelerated T1D in animal models, the observation that different antibiotics were associated with dramatically different T cell phenotypes may indicate distinct environment-driven endotypes. Similarly, regional differences in commensal microbiota composition may be considered a distinct category of environmental-driven endotype. However, mutual regulation and feedbacks make it difficult to draw a clear line between genetics- and environment-driven endotypes as HLA differences have been reported to influence the gut microbiome (193). A recent report indicated that individuals carrying at-risk HLA haplotypes seem to have a distinct core microbiome and beta diversity compared to the general population (194). This raises an interesting question: to what degree is the dysbiotic gut environment shaped by the HLA variants? Future investigations in this direction may provide new insights into the rate at which at-risk individuals become diabetic.

Another related area worthy of further exploration is the role of gut microbiota in shaping the diabetogenic T cell repertoire. As discussed in this review, our own work has recently identified a connection between the gut microbiota and thymocyte development (131). Our data indicate that antigens derived from the gut microbiota can be transferred to the thymus *via* migratory DCs trafficking between the gut and the thymus, resulting in the expansion of developing T cells specific for the microbiota. Selection of gut microbiota-specific T cell repertoire by the risk-conferring MHC variants and the propensity of this T cell repertoire for Th1/2/17 and Treg differentiation in the periphery in response to the microbiota may provide a mechanistic explanation for the distinct microbiome observed in individuals carrying at-risk HLA haplotypes. Furthermore, how this process regulates T cell response toward commensals carrying mimics of diabetogenic epitopes would be insightful as well.

**Table 1:** Association of Gut Commensal Bacteria with T1D incidence and proposed mechanisms.

Phylum	Family	Genus/Species	T1D correlation	Potential mechanism	References
Bacteroidetes	Bacteroidaceae	*B. stercoris*	Positive	Molecular mimicry	(27)
		*B. vulgatus*	Positive	Molecular mimicry	(17, 28)
	Tannerellaceae	*P. distasonis*	Positive	Molecular mimicry	(13, 29)
	Prevotellaceae	*Prevotella* spp.	Negative	SCFA production	(19, 21)
Firmicutes	Clostridiaceae	*SFB*	Negative	IL-22 production by gut ILC3s, Th17 skewing	(30–32)
		*F. prausnitzii* (Cluster IV)	Negative	SCFA production	(18, 33)
		*Roseburia* spp. (Cluster XIVa)	Negative	SCFA production	(17, 18, 34)
	Lactobacilliaceae	*L. rhamnosus*	Negative	SCFA production, claudin-3 upregulation, gut barrier integrity	(17, 35, 36)
		*L. johnsonii*	Negative	Th17 skewing, gut-barrier integrity	(37, 38)
Actinobacteria	Bifidobacteriaceae	*B. longum*	Negative	SCFA production, gut-barrier integrity	(17, 39)
		*B. breve*	Negative	SCFAs, RegIIIγ upregulation	(13, 17, 40)
		*B. dentium*	Negative	Acetate production, mucin upregulation	(17, 41)
Verrucomicrobia	Akkermansiaceae	*A. muciniphila*	Negative	RegIIIγ and mucin upregulation	(42–44)
Fusobacteria	Fusobacteriaceae	*L. goodfellowii*	Positive	Molecular mimicry	(45)

## Author contributions

YL and SM contributed equally to this work. YL, SM, and MB wrote and edited the article. All authors contributed to the article and approved the submitted version.

## Funding

This study was supported by the National Institute of Diabetes and Digestive and Kidney Diseases (grant R01-DK114456) and National Institute of Allergy and Infectious Diseases (R01-AI136963).

## Conflict of interest

The authors declare that the research was conducted in the absence of any commercial or financial relationships that could be construed as a potential conflict of interest.

## Publisher’s note

All claims expressed in this article are solely those of the authors and do not necessarily represent those of their affiliated organizations, or those of the publisher, the editors and the reviewers. Any product that may be evaluated in this article, or claim that may be made by its manufacturer, is not guaranteed or endorsed by the publisher.
